# Profiles of Burnout, Coping Strategies and Depressive Symptomatology

**DOI:** 10.3389/fpsyg.2020.00591

**Published:** 2020-04-02

**Authors:** Juan Pedro Martínez, Inmaculada Méndez, Cecilia Ruiz-Esteban, Aitana Fernández-Sogorb, José Manuel García-Fernández

**Affiliations:** ^1^Department of Developmental Psychology and Education, University of Murcia, Murcia, Spain; ^2^Department of Developmental Psychology and Didactics, Faculty of Education, University of Alicante, Alicante, Spain

**Keywords:** emotions, psychological processes, organizational context, well-being, empirical evidence

## Abstract

Burnout syndrome is has been associated with mental health problems such as depression, anxiety, and stress. Given this fact, some teachers implement various coping strategies for emotional control that are not always functional to mitigate such difficulties. Accordingly, this study aimed to identify different burnout profiles that vary in the levels of the three underlying dimensions: depersonalization (DE), emotional exhaustion (EE), and personal accomplishment (PA). Further, this study aimed to examine whether there are significant differences in depressive symptomatology, coping strategies, and the quality of interpersonal relationships at school between teachers with varying burnout profiles. The Maslach Burnout Inventory (MBI), Zung Self-Rating Depression Scale (SDS), Coping with Stress Questionnaire, and a questionnaire that measured sociodemographic characteristics were administered to 215 teachers (men: 42.8%) who were recruited from various secondary schools. Cluster analysis identified three different burnout profiles: groups of teachers with a predominance of (a) low levels of EE and high levels of PA, (b) high levels of EE and DE, and (c) low levels of DE and PA. The results revealed that there were significant differences in coping strategies, depressive symptomatology, and the quality of interpersonal relationships at school between teachers with different burnout profiles. These results have important implications for educational professionals. Specifically, the findings underscore the need for prevention and intervention programs that enhance teachers’ emotional skills, especially their ability to cope with exhaustion. These skills will alleviate their depression and consequently offer both teachers and students a conducive learning environment.

## Introduction

A negative work environment can cause physical, psychological, and occupational problems. Specifically, work exhaustion, occupational stress, and job burnout can have significant negative effects. Accordingly, in their systematic review, Salvagioni et al. (2017) noted that past studies have shown that burnout has several adverse effects on the well-being and health of employees. Specifically, burnout emerged as a significant predictor of several physical (e.g., hypercholesterolemia, type 2 diabetes, coronary heart disease, hospitalization for cardiovascular disorders, musculoskeletal pain, prolonged fatigue, headaches, gastrointestinal issues, respiratory problems), psychological (e.g., insomnia, depressive symptoms, hospitalization for mental disorders, psychological ill-health symptoms), and occupational (e.g., job dissatisfaction, absenteeism, new disability pension, job demands) problems.

The term “burnout syndrome” was first coined by Freudenberger (1974). Currently, the International Classification of Diseases (ICD-11), which is published by the World Health Organization [WHO] (2019), considers burnout to be a syndrome that is related to chronic work stressors that have not been successfully handled. The symptoms of burnout include the following: exhaustion or a lack of energy, reduced professional efficiency, and negative or cynical feelings about work. Therefore, burnout is a three-dimensional syndrome that is experienced by professionals whose jobs require direct interactions with others. According to Maslach and Jackson (1986), this syndrome consists of three characteristic dimensions: emotional exhaustion (EE) (i.e., tiredness and fatigue, which can be manifested physically and psychologically), depersonalization (DE) (i.e., negative, cold, and distant attitudes toward the beneficiaries of work), and low levels of personal accomplishment (PA) (i.e., negative perceptions of oneself and one’s work and poor work performance, which result from avoiding personal and professional relationships).

In Spain, according to data from the National Statistics Institute (2018), the average work stress for both sexes is 4.18 (*SD* = 1.69). In terms of the levels of job stress, Spain ranks third among European countries. Teaching is a stressful profession, especially for beginning teachers (Harmsen et al., 2019). Different groups of teaching professionals are exposed to different types of physical and psychological risks (Gallardo-López et al., 2019). Several studies have shown that the teacher-student relationship is associated with the well-being of teachers. Consequently, student problem behaviors are associated with greater exhaustion and decreased enthusiasm among teachers (Nizielski et al., 2013; Aldrup et al., 2018; Aparisi et al., 2019), which in turn can cause them to abandon the teaching profession (Skaalvik and Skaalvik, 2018; Chambers Mack et al., 2019). The stress that teachers experience can also have negative effects on students (e.g., low levels of student satisfaction) (Ramberg et al., 2019). DE can result in low levels of professional consciousness and PA and cause teachers to be irritable, cynical, and critical; these factors involve psychological problems that can adversely affect the teaching-learning process (Yin, 2015). EE is associated with work overload, interpersonal conflicts, negative feedback, and low levels of social support, autonomy, and job satisfaction (Carlotto and Câmara, 2019; Molero et al., 2019).

The question of whether burnout is a type of depression or a different phenomenon has been the subject of controversy, especially because the two conditions share similar characteristics (e.g., a loss of interest, impaired concentration). Accordingly, in their systematic review, Bianchi et al. (2015) noted that the final stage of burnout is typically correlated with depressive symptoms. Koutsimani et al. (2019) conducted a systematic review and meta-analysis and found that burnout and depression as well as burnout and anxiety are robust and independent constructs that (a) share a few common characteristics, (b) are interconnected, and (c) can develop in tandem. Therefore, burnout is associated with both depression and anxiety.

Coping strategies are used when the demands of a stressful situation exceed individual resources. These strategies entail behavioral and cognitive efforts that aim to reduce or help an individual tolerate specific internal and/or external demands (Lazarus and Folkman, 1984). An individual may use various adaptive and maladaptive strategies to cope with stress (Sandín and Chorot, 2003). There are two types of coping strategies: direct or action-focused coping (i.e., they focus on modifying the source of stress and solving problems) and indirect or emotion-centered coping (i.e., they focus on regulating the emotional response to stress, avoiding the problem situation by engaging in other distracting activities, and seeking social support strategies. The main coping strategies that have been identified by Lazarus and Folkman (1984) are as follows: confrontation, distancing, self-control, seeking social support, accepting responsibility, escape-avoidance, problem solving, and positive reappraisal.

Several studies have found that coping strategies are directly related to burnout among teachers (Guerrero, 2003; Doménech and Gómez, 2010; Sharplin et al., 2011; Carson et al., 2012; Shin et al., 2014; Martínez, 2015; García-Arroyo and Osca, 2017, 2019; Dalcin and Carlotto, 2018; Yin et al., 2018). Coping strategies have a direct influence on the consequences of burnout. Coping strategies are negatively related to EE and cynicism and positively related to PA (Yin et al., 2018; García-Arroyo and Osca, 2019). DE is associated with the use of denial, mental disconnection, and avoidance. Thus, avoidance is frequently used by individuals with burnout syndrome. Avoidance is related to distancing, which in turn is indicative of a lack of commitment among teachers, and consequently, poor educational quality (Morán, 2009; Martínez, 2015; Yin et al., 2018; García-Arroyo and Osca, 2019). Teachers who experience high levels of EE and DE use coping strategies that necessitate passive acceptance, and they do not search for effective solutions that can help them manage stressful situations in the workplace. A high degree of PA is associated with the frequent use of strategies such as planning, active coping, seeking instrumental and social support and positive reappraisal (Guerrero, 2003; Doménech and Gómez, 2010; Martínez, 2015).

This study aimed to identify different burnout profiles that vary in the levels of the three underlying dimensions (i.e., DE, EE, and PA). Further, we sought to examine if teachers with varying burnout profiles significantly differ in depressive symptomatology, coping strategies, and the quality of interpersonal relationships at school.

## Materials and Methods

### Participants

A random sample of conglomerates (i.e., different geographical areas within the region of Murcia in Spain) was used. Specifically, an average of 15 participants was recruited from each of 20 randomly selected public and private/semiprivate educational institutions in rural and urban areas. The sample consisted of 300 teachers, and they taught grades 1–4 of Obligatory Secondary Education. However, 85 participants (28.33%) were excluded either because they submitted questionnaires containing erroneous or missing responses or because they did not wish to participate in the study. The final sample consisted of 215 teachers who were recruited from different geographical areas within the Region of Murcia (public institutions = 73.5%, private/semi-private institutions = 26.5%). Their ages ranged from 30 to 65 years (*M* = 44.89, *SD* = 9.36), and 42.8% of them were men.

### Design and Procedure

After we obtained the requisite permissions of the school authorities, we asked the teachers to complete the self-administered questionnaires within the school premises. The researchers informed them about the objectives of the study and the instruments that they would be required to respond to. They participated on a voluntary and anonymous basis, and their responses were kept confidential.

The study protocol was approved by the Ethic Committee for Clinic Investigations of the University of Murcia. This study was conducted in accordance with approved guidelines and the Declaration of Helsinki.

### Instruments

Burnout was assessed using the Spanish adaptation (Seisdedos, 1997) of the Maslach Burnout Inventory (MBI), which has been developed by Maslach and Jackson (1986). It consists of 22 items, and responses are recorded on a rating scale (*0* = *never, 6* = *everyday*). The test consists of three dimensions: emotional exhaustion -EE- (e.g., I feel frustrated by my job), depersonalization –DE- (e.g., I don’t really care about what happens to some recipients), and personal accomplishment –PA- (e.g., I deal very effectively with the problems of my recipients). The Cronbach’s alpha coefficients of the EE, DE, and PA dimensions were 0.90, 0.79, and 0.71 in the original validation study, respectively (Maslach and Jackson, 1986). In this study, the alpha coefficient of the total scale was 0.76.

Stress-related coping strategies were assessed using the questionnaire that has been developed by Sandín and Chorot (2003). It consists of 42 items, each of which requires responses to be recorded on a rating scale (*0* = *never, 4* = *usually*). This assessment consists of seven subscales: seeking social support (e.g., I asked a relative or friend for advice to deal with the problem in a better manner), overt emotional expression (e.g., I behaved in a hostile way toward others), religion (e.g., I went to church to pray for the problem to be solved), problem solving (e.g., I tried to analyze the causes of the problem in order to address it), avoidance (e.g., I tried to not think about the problem), negative self-targeting (e.g., I realized that I could not do anything to solve the problem), and positive reappraisal (e.g., I tried to get something positive out of the problem). In the original validation study, the Cronbach’s alpha coefficients of the seven subscales ranged from 0.64 to 0.92, and the average value was 0.79 (Sandín and Chorot, 2003). In this study, the alpha coefficient of the total scale was 0.81.

To measure the behavioral symptoms of depressive disorder, the Self-Rating Depression Scale (SDS) (Zung, 1965) was used. It consists of 20 items, each of which requires responses to be recorded on a rating scale *(1* = *rarely or never, 4* = *most of the time or always)*. The Cronbach’s alpha coefficients of this scale have ranged from 0.79 to 0.92 (Zung, 1965). In this study, the scale’ alpha coefficient was 0.83. The following is a sample scale item: “I feel downhearted and blue.”

The following sociodemographic characteristics were also assessed: sex (male/female), age, type of school (public/private/semi-private), geographical location (urban/rural), the quality of interpersonal relationships at school (i.e., with students, teaching staff members, and the management team; response scale: *1* = *rewarding, 4* = *frustrating*), and salary satisfaction (yes/no).

### Data Analysis

To identify burnout profiles, quick cluster analysis was conducted (Hair et al., 1998). The profiles were defined based on the differential combinations of the three dimensions that the MBI assesses: DE, EE, and PA. The following criterion was used to ascertain the optimal number of clusters: the maximization of intercluster differences so that the largest number of groups with differential combinations of the burnout dimensions are identified. In addition, the theoretical feasibility and psychological significance of each group that represented a specific burnout profile were also considered. After identifying different burnout profiles through cluster analysis, analysis of variance (ANOVA) was conducted to examine group differences in depressive symptomatology, stress-related coping strategies, and the quality of the interpersonal relationships at school. Partial eta squared (η*_*p*_*^2^) values were computed to ascertain the magnitude of the emergent group differences (i.e., effect size). *Post hoc* tests (i.e., Bonferroni method) were conducted to further identify the exact groups that were significantly different. Cohen’s *d* was computed to ascertain the magnitude of the observed differences, and the values were interpreted as being indicative of a small (0.20 ≤ *d* ≤ 0.49), moderate (0.50 ≤ *d* ≤ 0.79), or large (*d* ≥ 0.80) effect size (Cohen, 1988). The data were analyzed using Statistical Package for the Social Sciences version 23.0.

## Results

**FIGURE 1:**
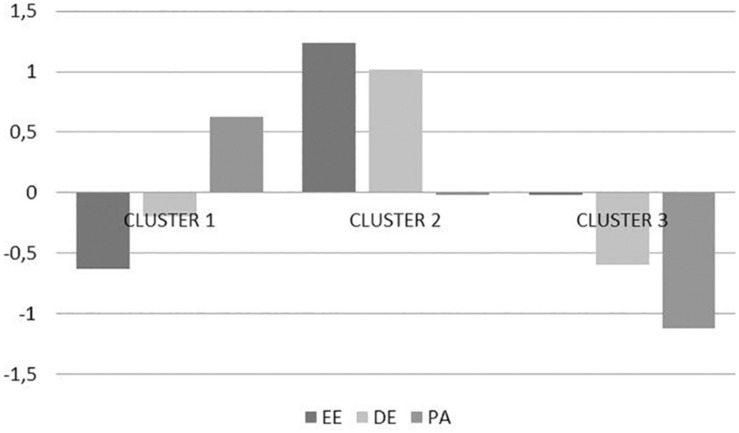
Graphical representation of the three-cluster model. Cluster 1 (low EE and high PA), cluster 2 (high EE and high DE), and cluster 3 (low DP and low PA).

Figure 1 depicts the following three emergent clusters: a first group of 104 teachers (48.37%) characterized by low EE and high PA (group 1); a second group of 54 teachers (25.12%) characterized by high EE and DE (group 2); and a third group of 57 teachers (26.51%) characterized by low DE and PA (group 3).

Analysis of variances revealed that there were significant group differences in coping strategies, depressive symptomatology, and the quality of interpersonal relationships at school (see Table 1).

**TABLE 1:** Means and standard deviations for the three groups differing in the dimensions of burnout and partial eta squared (η*p*^2^) values for each dimension of coping strategies, depressive symptomatology, and the quality of interpersonal relationships at school.

	Group 1		Group 2		Group 3		Significance		
*Dimensions*	*M*	*SD*	*M*	*SD*	*M*	*SD*	*F*_(2,121)_	*P*	*η_*p*_^2^*
Search for social support	15.28	6.37	15.13	5.84	12.89	5.60	3.12	00.046	0.03
Overt emotional expression	6.53	3.31	7.83	3.17	7.19	2.92	3.08	00.048	0.03
Religion	6.37	6.80	5.78	6.35	5.32	6.41	0.48	00.614	0.01
Focus on solving the problem	18.09	3.90	16.17	4.21	15.58	3.96	8.56	< 0.001	0.08
Avoidance	10.23	4.35	10.78	3.55	10.63	3.17	0.41	00.660	0.01
Negative self-targeting	6.14	3.70	8.30	3.65	7.53	3.42	6.98	00.001	0.06
Positive reappraisal	15.84	3.62	14.02	4.11	14.54	3.58	4.86	00.009	0.04
Depressive symptomatology	38.88	6.96	52.27	11.41	45.86	8.97	42.70	< 0.001	0.29
Student relations	1.82	0.72	2.39	0.87	2.16	0.72	10.68	< 0.001	0.09
Teacher relations	1.71	0.66	2.02	0.83	1.95	0.81	3.61	00.029	0.03
Relations with the management team	1.87	0.75	2.52	1.01	2.07	0.70	11.547	< 0.001	0.10
Salary satisfaction	1.44	0.63	1.61	0.56	1.46	0.68	1.370	00.256	0.01

*Cluster 1 (low EE and high PA), cluster 2 (high EE and high DE), and cluster 3 (low DP and low PA).*

The *post hoc* comparisons revealed that group 1 (low EE and high PA) obtained significantly higher scores on the seeking social support subscale than group 3 (low DE and PA), and the effect size was small (*p* < 0.05; *d* = 0.39). Similarly, group 2 (high EE and DE) also obtained significantly higher subscale scores than group 3 (low DE and PA), and the effect size was small (*p* < 0.05; *d* = 0.39).

The *post hoc* comparisons revealed that group 1 (low EE and high PA) obtained significantly lower scores on the overt emotional expression subscale than group 2 (high EE and DE), and the effect size was small (*p* < 0.05; *d* = 0.40).

With regard to the religion and avoidance subscales, no significant group difference emerged.

The *post hoc* comparisons revealed that group 1 (low EE and high PA) obtained significantly higher scores on the problem solving subscale than group 2 (high EE and DE), and the effect size was small (*p* < 0.05; *d* = 0.48). In addition, group 1 (low EE and high PA) also obtained significantly higher subscale scores than group 3 (low DE and PA), and the effect size was moderate (*p* < 0.001; *d* = 0.64).

The *post hoc* comparisons revealed that group 1 (low EE and high PA) obtained significantly lower scores on the negative self-targeting subscale than group 2 (high EE and DE), and the effect size was moderate (*p* < 0.001; *d* = 0.59).

The *post hoc* comparisons revealed that group 1 (low EE and high PA) obtained significantly higher scores on the positive reappraisal subscale than group 2 (high EE and DE), and the effect size was small (*p* < 0.05; *d* = 0.48).

With regard to depressive symptomatology, *post hoc* comparisons revealed that group 2 (high EE and DE) obtained significantly higher scores than group 1 (low EE and high PA), and the effect size was large (*p* < 0.001; *d* = 1.53). Relatedly, group 3 (low DE and PA) scored significantly higher than group 1 (low EE and high PA), and the effect size was large (*p* < 0.001; *d* = 0.90). Finally, group 2 (high EE and DE) scored significantly higher than group 3 (low DE and PA), and a moderate effect size emerged for this group difference (*p* < 0.001; *d* = 0.63).

The *post hoc* comparisons of the quality of the relationships that teachers shared with students revealed that group 1 (low EE and high PA) obtained significantly lower scores than group 2 (high EE and DE), and the effect size was moderate (*p* < 0.001; *d* = 0.74). Similarly, group 1 (low EE and high PA) scored lower than group 3 (low DE and PA), and the effect size was small (*p* < 0.05; *d* = 0.47).

The *post hoc* comparisons of the quality of the relationships that teachers shared with other staff members revealed that group 1 (low EE and high PA) obtained significantly lower scores than group 2 (high EE and DE), and the effect size was small (*p* < 0.05; *d* = 0.43).

The *post hoc* comparisons of the quality of the relationships that teachers shared with the management team revealed that group 1 (low EE and high PA) scored significantly lower than group 2 (high EE and DE), and the effect size was moderate (*p* < 0.001; *d* = 0.77). Similarly, group 3 (low DE and PA) also obtained lower scores than group 2 (high EE and DE), and the effect size was moderate (*p* < 0.05; *d* = 0.52).

No significant group difference in salary satisfaction was found.

## Discussion

This study had two objectives. The first objective was to identify different burnout profiles that vary in the levels of the underlying dimensions (i.e., EE, DE, and PA). Using cluster analysis, three burnout profiles were identified: groups of teachers who were characterized by low EE and high PA (group 1), high EE and DE (group 2), and low DE and PA (group 3). With regard to the second objective, the results revealed that there were significant group differences in coping strategies, depressive symptomatology, and the quality of interpersonal relationships at school. These findings offer support to the contention that different burnout profiles exist. Further, the present findings enhance our understanding of the relationships that the three burnout profiles share with coping strategies, depressive symptomatology, and the quality of interpersonal relationships at school. Taken together, the results revealed that group 1 (low EE and high PA) obtained higher scores on the seeking social support (i.e., sharing feelings with family members or friends to feel reassured), problem solving (i.e., identifying the causes of the problem and developing an action plan), and positive reappraisal (i.e., focusing on the positive aspects of a negative situation and realizing that there are more important things in life) subscales than groups 2 and 3. On the other hand, group 2 (high EE and DE) obtained higher scores on the overt emotional expression (i.e., moodiness, bad behavior, or hostility) and negative self-targeting (i.e., self-doubt, resignation, or helplessness) subscales as well as measures of depressive symptomatology and the quality of interpersonal relationships at school (i.e., with students, teachers, and the management team) than groups 1 and group 3. These results concur with past findings that coping has a direct influence on the consequences of burnout (Guerrero, 2003; Doménech and Gómez, 2010; Sharplin et al., 2011; Carson et al., 2012; Shin et al., 2014; Martínez, 2015; García-Arroyo and Osca, 2017, 2019; Dalcin and Carlotto, 2018; Yin et al., 2018). Specifically, high levels of EE are positively associated with greater use of negative self-targeting and overt emotional expression. In addition, high levels of DE are also associated with greater use of negative self-targeting. Similarly, the use of coping strategies that focus on problem solving is related to lower levels of stress and higher levels of social support, and consequently, better physical and psychological health (Guerrero, 2003; Doménech and Gómez, 2010; Martínez, 2015; Dalcin and Carlotto, 2018; Yin et al., 2018; García-Arroyo and Osca, 2019).

The present finding that burnout is associated with depression is consistent with Koutsimani et al. (2019) conclusions. Similarly, the findings that emerged for group 2 (and to some extent, group 3) underscore the need to subject the respective group members to a clinical evaluation so that their diagnosis can be confirmed after eliminating other possible conditions (e.g., anxiety; Koutsimani et al., 2019). This will play a particularly important role in the provision of timely interventions. Indeed, the members of group 2 were educational professionals, who are known to experience high levels of stress, which can impact the teaching and learning process and increase their likelihood of developing health problems or absenteeism (Carlotto and Câmara, 2019; Chambers Mack et al., 2019; Gallardo-López et al., 2019; Ramberg et al., 2019).

It is necessary to promote interpersonal relationships, social support networks, and emotion regulation among teachers with high EE and DE because they obtained high scores on the overt emotional expression (i.e., the expression of anger or rage) subscale. When taken together with the finding that this group of teachers reported greater frustration with their interpersonal relationships at school (with students, staff members, and the management team), it appears that their negative emotions were reinforced (i.e., negative self-targeting) by their inability to solve their problems; this in turn may have resulted in negative and even depressive feelings. In contrast, teachers with low EE and high PA were more likely to seek social support, and this allowed them to be a part of a support network within which they could feel supported and listened to when they encountered problems. This also helped them address the problem (i.e., focusing on problem solving) and either focus on the positive aspects of the negative situation or realize that there are more important things in life (i.e., positive reappraisal). In this manner, positive reappraisals can help teachers adaptively alter the outcomes of an otherwise negative situation (Guerrero, 2003; Morán, 2009; Martínez, 2015; Dalcin and Carlotto, 2018). These findings facilitate the identification of the coping strategies that should be nurtured among teachers.

It is essential to consider the important role that emotion regulation plays in coping with stressful situations. Indeed, past studies have shown that high levels of emotional intelligence predict better psychological and emotional adjustment among teachers (Lischetzke and Eid, 2003; Mearns and Cain, 2003; Biglan et al., 2013; Nizielski et al., 2013; Ghanizadeh and Royaei, 2015; Yin, 2015; Cabello and Fernández-Berrocal, 2016; Rey et al., 2016; Fernández-Berrocal et al., 2017; Grandey and Melloy, 2017; Yin et al., 2018; Schoeps et al., 2019). Teachers with poor emotional intelligence tend to report higher levels of EE, DE, anxiety, depression, and burnout (Martínez-Monteagudo et al., 2019). In this manner, improving emotion regulation is likely to be accompanied by an increase in the quality and number of social relationships at work, empathy, and job satisfaction (Brackett et al., 2010; Ghanizadeh and Royaei, 2015; Yin, 2015; Zysberg et al., 2017; Yin et al., 2018). Puertas Molero et al. (2019) conducted a systematic review of studies that have examined the role of emotional intelligence in stress among teachers and concluded that past findings underscore the importance of developing the emotional skills of educational professionals, including those who work in universities. Emotional skills can help them regulate their emotions and improve their day-to-day decision making within the school environment. These changes are likely to promote physical and mental health and consequently improve teaching practices, the institutional climate, and most importantly, the quality of education. Accordingly, it is necessary to enhance the emotional intelligence of teachers who belong to group 2 (high EE and DE) because superior emotional and self-regulation skills are essential to the alleviation of high degrees of exhaustion. Similarly, it is necessary to improve the following, especially among teachers who belong to group 2: the illusion at work (Dalcin and Carlotto, 2018); the perceived effectiveness of teachers (i.e., individual and collective) and job satisfaction (Guidetti et al., 2018; Minghui et al., 2018; Molero et al., 2019; Von Der Embse et al., 2019); welfare and commitment (Skaalvik and Skaalvik, 2018); adaptive working conditions (Chambers Mack et al., 2019; Eurofound, 2019); the social support of coworkers; effective support to meet family demands and prevent role conflict between work and family demands (i.e., personal exhaustion that results from work overload can worsen the family situation; e.g., it can reduce the quality of care that is provided to family members); and time management skills (De Carlo et al., 2019; Eurofound, 2019; World Health Organization [WHO], 2019). It is also important to reduce role ambiguity by clarifying the responsibilities of each professional (Carlotto and Câmara, 2019; De Carlo et al., 2019; Eurofound, 2019; Molero et al., 2019) and minimize the tediousness of administrative procedures (Carlotto and Câmara, 2019). It is also essential to proactively identify and gratify student needs so that the emergence or worsening of problem situations can be prevented. Problem situations can cause stress (Nizielski et al., 2013; Kaya and Altınkurt, 2018), exhaustion, a loss of enthusiasm (Aldrup et al., 2018; Aparisi et al., 2019), or even the abandonment of the teaching profession (Skaalvik and Skaalvik, 2018; Chambers Mack et al., 2019) among teachers. Most importantly, it is essential for policy makers to reconceptualize the roles of teachers by realistically restructuring their workload and clarifying their responsibilities (Carlotto and Câmara, 2019; De Carlo et al., 2019; Molero et al., 2019).

Finally, since burnout has a wide range of effects on the well-being and health of employees (Salvagioni et al., 2017), the importance of good mental health should be emphasized by making it possible for individuals to realize their potential, cope with stress, and work productively According to the Mental Health Action Plan 2013–2020 (World Health Organization [WHO], 2013), it is necessary for global strategies that aim to promote mental health and prevent mental disorders in the workplace to focus on the establishment of healthy living and working conditions (e.g., organizational measures, stress management plans). It is also necessary to identify and treat disorders that are caused by the harmful effects of alcohol and drug intake (both psychoactive and non-psychoactive drugs) as well as prevent suicide. These measures will play an important role in improving mental health in the workplace.

One of the limitations of the present study pertains to the use of self-reported data; specifically, distorted and socially desirable responses may have biased the present findings. Future research studies should examine other variables such as addictions to new technologies or drugs, self-concept or self-esteem, prior physical and mental health status, and medication consumption (Bianchi et al., 2015) in relation to burnout. They should also adopt longitudinal research designs and investigate the neurobiological mechanisms that underlie burnout (Koutsimani et al., 2019).

## Data Availability Statement

The datasets generated for this study are available on request to the corresponding author.

## Ethics Statement

The studies involving human participants were reviewed and approved by the study protocol and the Ethic Committee for Clinic Investigations of the University of Murcia (July 02, 2019). The patients/participants provided their written informed consent to participate in this study.

## Author Contributions

JM, IM, CR-E, AF-S, and JG-F contributed to the conception and design of the review. CR-E and AF-S applied the search strategy. All authors applied the selection criteria, completed the bias-risk assessment, analyzed and interpreted the data, wrote the manuscript, and edited this manuscript.

## Conflict of Interest

The authors declare that the research was conducted in the absence of any commercial or financial relationships that could be construed as a potential conflict of interest.
